# Reproductive traits of the symbiotic pea crab *Austinotheres angelicus* (Crustacea, Pinnotheridae) living in *Saccostrea palmula* (Bivalvia, Ostreidae), Pacific coast of Costa Rica

**DOI:** 10.3897/zookeys.457.7851

**Published:** 2014-11-25

**Authors:** Carolina Salas-Moya, Sebastián Mena, Ingo S. Wehrtmann

**Affiliations:** 1 Escuela de Biología, Universidad de Costa Rica, 11501-2060 San José, Costa Rica; 2 Unidad de Investigación Pesquera y Acuicultura (UNIP) del Centro de Investigación en Ciencias del Mar y Limnología (CIMAR), Universidad de Costa Rica, 11501-2060 San José, Costa Rica

**Keywords:** Central America, fecundity, reproductive output, symbiosis

## Abstract

Pea crabs of the family Pinnotheridae exhibit a symbiotic life style and live associated with a variety of different marine organisms, especially bivalves. Despite the fact that pea crabs can cause serious problems in bivalve aquaculture, the available information about the ecology of these crabs from Central America is extremely limited. Therefore, the present study aimed to describe different reproductive features of the pinnotherid crab *Austinotheres angelicus* associated with the oyster *Saccostrea palmula* in the Golfo de Nicoya, Pacific coast of Costa Rica. Monthly sampling was conducted from April to December 2012. Average carapace width (CW) of the 47 analyzed ovigerous females was 7.62 mm. The species produced on average 2677 ± 1754 recently -extruded embryos with an average volume of 0.020 ± 0.003 mm^3^; embryo volume increased during embryogenesis by 21%, but did not vary significantly between developmental stages. Brood mass volume varied greatly (between 11.7 and 236.7 mm^3^), and increased significantly with female CW. Females invested on average 76.7% (minimum: 21.7%; maximum: 162.8%) of their body weight in brood production, which confirms a substantially higher energy allocation for embryo production in pinnotherid crabs compared to free-living decapods.

## Introduction

Symbiotic relationships in marine organisms are a well-documented phenomenon (Roughgarden 1975, Dos Santos-Alves and Pezzuto 1998, Boltaña and Thiel 2001, Thiel and Baeza 2001, Sotka 2005, Baeza 2007, Glynn 2013). The evolution of these symbiotic associations is shaped by cost and benefit aspects for both partners (Roughgarden 1975, Baeza 2007), but the role of ecological features responsible for the evolution of this lifestyle remains unclear (Baeza 2007). According to different studies (Baeza 2007, Ory et al. 2013), predation pressure is one of the principal forces driving the evolution of symbiotic associations in decapods. Small marine decapods commonly live associated with anemones, echinoderms and a variety of other invertebrates, which may serve as refuge from predation, food source or mating site (Criales 1984, Bauer 2004, Wirtz et al. 2009, Ory et al. 2013). However, studies on symbiotic decapods are still scarce, especially in those cases where environmental conditions hamper direct observations (Baeza 1999).

The Pinnotheridae is a highly diverse family, currently with 52 genera and more than 300 described species (Ng et al. 2008, De Grave et al. 2009, Palacios-Thiel et al. 2009). Members of this family are known to exhibit symbiotic relationships with numerous other invertebrates, including mollusks, polychaetes, echinoderms, brachiopods, and other decapods (Manning and Morton 1987, Feldmann et al. 1996, Baeza 1999, Lardies and Castilla 2001, McDermott 2006, Peiró and Mantelatto 2011, Trottier et al. 2012). The type of symbiotic relationships ranges from parasitism to commensalism, and pinnotherids live in a facultative and/or obligate association with their hosts (Silas and Alagarswani 1965, Schmitt et al. 1973, Stevens 1990, Hamel et al. 1999). Detailed information about many aspects of the association between pinnotherid crabs and their host remain to be studied (McDermott 2006, Peiró et al. 2011). Several studies addressed different reproductive aspects of these symbiotic crabs, including the morphology of the reproductive system (Becker et al. 2011, 2012, 2013), mating system (Dos Santos-Alves and Pezzuto 1998, Baeza 1999, Peiró et al. 2013), intraspecific latitudinal effects on different reproductive attributes (Lardies and Castilla 2001), as well as fecundity (Báez and Martínez 1976, Hines 1992, Cabrera Peña et al. 2001, Lardies and Castilla 2001). Our knowledge about energy allocation in embryo production of pinnotherid crabs is scarce; however, results obtained from several species of pea crabs clearly demonstrated that brood masses of these crabs are extraordinarily large in relation to female body size when compared to free-living decapods (Hines 1992, Lardies and Castilla 2001). This high investment in brood production is related to two unique features of the pea crabs (Hines 1992): (1) their ovaries cover not only the cephalothorax, but extend into the abdomen; (2) the exoskeleton of females is not well calcified, which allows that the body is distensible during yolk accumulation.

**Figure 1.**
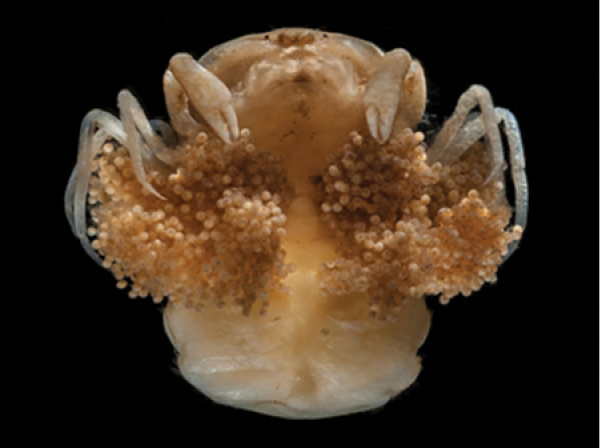
Ovigerous female of *Austinotheres angelicus* from the Golfo de Nicoya, Pacific coast of Costa Rica.

The pinnotherid crab *Austinotheres angelicus* Lockington, 1877 (Fig. 1) is distributed along the Pacific coast from the Gulf of California, Mexico, to Colombia, and lives in association with oysters (Campos 2002). Life history aspects of this species are virtually unknown, and therefore, the present study aimed to describe fecundity, embryo volume, and reproductive output of *Austinotheres angelicus* living in the oyster *Saccostrea palmula* (Carpenter, 1857) in the Golfo de Nicoya, Pacific coast of Costa Rica. The results of this study will broaden our knowledge of the evolution of reproductive strategies in decapods adapted to live in association with other marine invertebrates.

## Methods

### Field work

**Figure 2.**
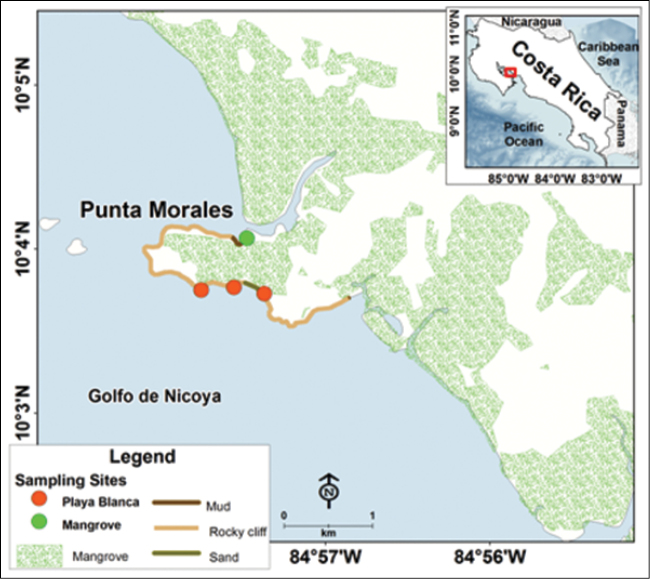
Sampling sites in Punta Morales, Golfo de Nicoya, Pacific coast of Costa Rica.

The study site was Punta Morales, Golfo de Nicoya (Fig. 2), located at the Pacific coast of Costa Rica. The location is a sandy beach with a rocky intertidal zone, surrounded by mud flats and mangrove swamps (Rojas and Vargas 2008). Monthly sampling was carried out between April and December 2012; collections were conducted during diurnal low tides. During each sampling, 30 oysters (*Saccostrea palmula*) were collected, preserved in ethanol (70%), and transported to the laboratory of the Unidad de Investigación Pesquera y Acuicultura (UNIP) of the Centro de Investigación en Ciencias del Mar y Limnología (CIMAR), Universidad de Costa Rica, in San José. Voucher specimens of *Austinotheres angelicus* were deposited in the collections of the Museo de Zoología, Universidad de Costa Rica (MZUCR 3281-01, 3282-01, 3283-01, 3284-01, 3285-01, 3286-01, 3287-01, 3288-01, 3289-01).

### Laboratory analyses

Morphometric measurements of oysters (height, length, and thickness) were obtained with a digital caliper (± 0.01 mm); the three morphometric variables were multiplied in order to calculate the approximate volume of each oyster (OV). Each individual of *Saccostrea palmula* was carefully opened and inspected for associated pea crabs. The carapace length (CL: distance between distal part of the eye socket to the posterior margin of the carapace), carapace width (CW: distance between lateral margins of the carapace), abdomen length (AL: distance between posterior margin of the carapace to the distal part of the abdomen) and abdomen width (AW: distance between lateral margins of the abdomen) were measured with the aid of a Leica MS5 stereoscopic microscope equipped with a calibrated ocular micrometer.

The entire brood mass was detached from ovigerous females (n = 47), evenly distributed on a Petri dish, and photographed (Benq GH650). These images were analyzed subsequently with the program ImageJ® versión 1.46r to count the total number of embryos carried by each ovigerous female. Embryos were staged according to the following criteria (Wehrtmann 1990): Stage I, uniform yolk, no eye pigments observed; Stage II, eye pigments scarcely visible; Stage III, embryo with eye pigments clearly visible and fully developed. Embryo volume (EV) was estimated using the equation (1) proposed by Corey and Reid (1991):

EV = (*b* / 2)^2^ × *a* × *b* × π (1)

where *a* is the major diameter, and *b* is the perpendicular diameter. A total of 15 embryos were measured from each female, and the average embryo volume was multiplied by the total number of embryos per female to calculate brood mass volume.

Females and their separated brood masses were dried at 60 °C for 48 hours, and dry weights were measured to calculate the reproductive output (RO): dry weight of total brood mass per female / dry weight of females without brood mass (Hines 1988). The RO was estimated exclusively for females carrying recently extruded embryos (Stage I).

## Data analyses

The Kruskal-Wallis test was applied to detect possible differences in both brood numbers between the three embryonic stages, and embryo volume during embryogenesis. Simple linear regressions and the Pearson correlation coefficient were calculated to determine the relation between both host morphometry (OV: volume of the oyster) and CW of *Austinotheres angelicus* (considering exclusively females with recently -produced embryos), fecundity (exclusively embryos in Stage I) and the following morphometric variables of the female: CW, CL, AW, and AL; and finally brood mass volume and CW of ovigerous females. All statistical analyses were carried out with JMP® version 7.0.

## Results

**Figure 3.**
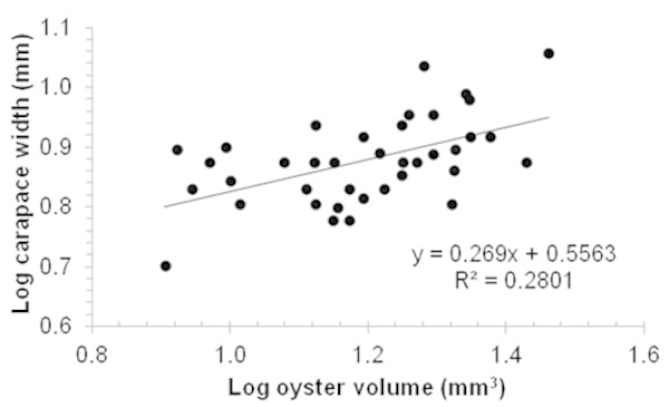
Relation between oyster volume (*Saccostrea palmula*) and carapace width of ovigerous females of *Austinotheres angelicus* (n = 38; exclusively females with recently produced embryos) in the Golfo de Nicoya, Pacific coast of Costa Rica.

**Figure 4.**
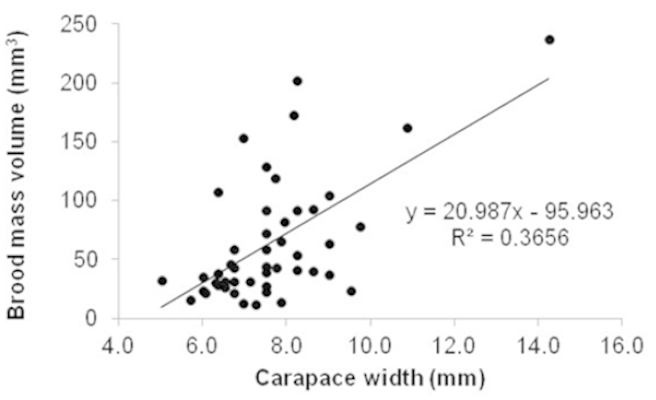
Relation between brood mass volume and carapace width of ovigerous females of *Austinotheres angelicus* (n = 47) in the Golfo de Nicoya, Pacific coast of Costa Rica.

The CW of the ovigerous female crabs averaged 7.62 mm, and ranged in size from 5.02 to 14.25 mm (Table 1). Mean fecundity was 2677 in Stage I and 4890 embryos in females with brood masses in Stage III (Table 1); however, mean embryo number did not vary significantly between developmental stages (χ^2^ = 2.57; gl = 2; p = 0.28). The CW of *Austinotheres angelicus* was significantly influenced by the volume of its host, *Saccostrea palmula* (t = 3.74; n = 38; p = 0.0006) (Fig. 3). Fecundity was positively related with all morphometric variables of *Austinotheres angelicus* (Table 2); the variable, which best explained the number of embryos was CW (Fig. 4). Brood mass volume varied between 11.70 mm^3^ (7.25 mm CW) and 236.70 mm^3^ (14.25 mm CW), and increased significantly with female CW (t = 5.09; n = 47; p < 0.001) (Fig. 4). Recently produced embryos (Stage I) had an average volume of 0.020 mm^3^, while those close to hatching (Stage III) measured 0.024 mm^3^ (Table 1); embryo volume increased during the embryogenesis by 21%, but did not vary significantly between stages (χ^2^ = 4.51; gl = 2; p = 0.10). The mean RO was 76.7 ± 28.90%, and values fluctuated between 21.7 and 162.8%.

**Table 1.** Fecundity, embryo volume during embryogenesis (Stage I–III) and carapace width of corresponding ovigerous females of *Austinotheres angelicus* (n = 47), Golfo de Nicoya, Pacific coast of Costa Rica.

Stage	N	Fecundity				Embryo volume (mm^3^)				Carapace width (mm)			
		Av	SD	Min	Max	Av	SD	Min	Max	Av	SD	Min	Max
I	35	2677	1764	550	7527	0.020	0.003	0.013	0.027	7.60	1.18	5.02	10.87
II	6	3029	2599	955	7900	0.023	0.005	0.017	0.031	7.27	1.21	5.70	9.00
III	6	4890	3176	830	8509	0.024	0.005	0.017	0.031	8.10	3.10	6.07	14.25

**Table 2.** Relation between morphometric features (CW, CL, AW, and AL) and fecundity in *Austinotheres angelicus* (n = 47) in the Golfo de Nicoya, Pacific coast of Costa Rica.

	CW	CL	AW	AL
**R^2^**	0.33	0.09	0.29	0.29
**T**	4.69	2.13	4.27	4.25
**P**	<0.001	0.04	0.001	<0.001

## Discussion

This is the first published report on reproductive aspects of *Austinotheres angelicus*. Cabrera Peña et al. (2001) studied the size composition and fecundity of *Juxtafabia muliniarum* (Rathbun, 1918) in the Pacific of Costa Rica; however, according to Campos and Vargas-Castillo (2014) they confused the species with *Austinotheres angelicus*. Regarding reproductive traits, Cabrera Peña et al. (2001) provided only average embryo numbers and stated that ovigerous females of this pea crab species were present throughout the study period (May 1998 to May 1999).

### Fecundity

Brood size increases with female body size have been well documented for pinnotherids (Hines 1992); not surprisingly, this study has found that small-sized species such as *Austinotheres angelicus* produce less offspring than substantially larger species (Table 3). The only other published data about reproductive features of *Austinotheres angelicus* (Cabrera Peña et al. 2001; reported as *Juxtafabia muliniarum*) indicate a slightly lower average fecundity than obtained in the present study (Table 3). This difference is probably related to the fact that our material contained larger females (as CW) as compared to those specimens analyzed by Cabrera Peña et al. (2001).

**Table 3.** Comparison of female size (CL and CW), fecundity, embryo volume during early embryogenesis, and reproductive output of different pinnotherids; host species and study sites are indicated. NA: no data available.

Species	CL (mm)	CL range (mm)	CW (mm)	CW range (mm)	Average fecundity	Fecundity range	Embryo volume (mm^3^)	RO (%)	Host	Study site	Reference
*Austinixa gorei*	NA	NA	6.80	6.30–8.90	NA	195–525	NA	NA	*Gilvossius setimanus* (Malacostraca, Callianassidae)	United States of America, northwest Point on Key Biscayne in Bear Cut	McDermott (2006)
Austinotheres angelicus*	5.60	4.00–7.60	6.87	4.90–9.40	2032**	680–3300	NA	NA	*Saccostrea palmula* (Bivalvia, Ostreidae)	Costa Rica, mangroves at Punta Morales, Pacific coast	Cabrera Peña et al. (2001)
*Austinotheres angelicus*	5.43	3.82–9.37	7.60	5.03–10.87	2677	550–8509	0.020	77	*Saccostrea palmula* (Bivalvia, Ostreidae)	Costa Rica, Punta Morales, Pacific coast	Present study
*Fabia subquadrata*	NA	NA	NA	NA	7560	NA	0.037	97	*Mytilus californianus* (Bivalvia, Mytilidae)	United States of America, Bodega Head, CA	Hines (1992)
*Pinnaxodes chilensis*	12.22	8.20–15.50	NA	NA	4553	2134–9456	0.048	70	*Loxechinus albus* (Echinoidea, Parechinidae)	Chile, Caleta Coloso	Lardies and Castilla (2001)
*Pinnaxodes chilensis*	16.39	10.75–20.00	NA	NA	8358	2376–15898	0.070	80	*Loxechinus albus* (Echinoidea, Parechinidae)	Chile, El Quisco	Lardies and Castilla (2001)
*Pinnaxodes chilensis*	18.59	17.40–20.30	NA	NA	8082	5045–15432	0.072	81	*Loxechinus albus* (Echinoidea, Parechinidae)	Chile, Mehuín	Lardies and Castilla (2001)
*Zaops ostreus*	NA	NA	NA	NA	5680	NA	0.092	66	*Crassostrea virginica* (Bivalvia, Ostreidae)	United States of America, Indian River, FL	Hines (1992)

* Reported as *Juxtafabia muliniarum*;

** Stage of embryonic development used to calculate fecundity is unknown.

### Brood loss

Brood loss is a well described phenomenon in decapods (for review: Kuris 1991). Our results did not reveal any embryo loss during the embryogenesis in *Austinotheres angelicus*; in fact, average embryo number in Stage I was higher than in Stage III (Table 1). This surprising result is explained by the fact that in our study specimens carrying embryos in Stage III were considerably larger than those with recently extruded embryos (Table 1). Lardies and Castilla (2001) hypothesized that the internal habitat of the host as well as the immobility of the commensal may effect reduction of embryo loss when they found low brood mortality rates in *Pinnaxodes chilensis* H. Milne Edwards, 1837, living as a commensal on a sea urchin. Our data seem to corroborate this hypothesis. However, additional studies with similar-sized females of *Austinotheres angelicus* carrying embryos in different developmental stages are necessary to answer the question whether decapods protected by its host show less or no brood mortality during embryogenesis.

### Embryo volume

The embryo volume of *Austinotheres angelicus* increased during the incubation period by 21%. This increase is relatively low when compared to other marine decapods living in association with other invertebrates: *Synalpheus yano* (Ríos & Duffy, 2007) in the sponge *Lissodendoryx colombiensis* Zea & van Soest, 1986; 118% (Hernáez et al. 2010); and, *Pinnaxodes chilensis* (H. Milne Edwards, 1837) in the sea urchin *Loxechinus albus* (Molina, 1782): ranging from 58 to 77% (Lardies and Castilla 2001). Lardies and Wehrtmann (1996) suggested that increased water uptake during embryogenesis might serve as a buffer against environmental changes outside the embryo. Therefore, low embryo volume increase might suggest relatively stable conditions for embryo development of *Austinotheres angelicus* in its host.

Embryo size has been considered as an indicator for energy content provided by the female (McEdward and Coulter 1987). Compared to other pinnotherids (Table 3), *Austinotheres angelicus* produced relatively small embryos, and the embryo volume (0.020 mm^3^) is considerably smaller than the average value (0.045 mm^3^) calculated for 35 brachyuran crab species (Hines 1992). Therefore, it is concluded that females of *Austinotheres angelicus* allocate a relatively small amount of energy per embryo. However, this low maternal investment per offspring is compensated by a relatively large brood mass, which is reflected by extraordinary high RO values (up to 162.8%). These findings corroborate results of previous studies with pea crabs (Hines 1992, Lardies and Castilla 2001), which revealed substantially higher reproductive investment compared to other free-living decapod species. According to Hines (1992), the following features might explain the high RO values in pinnotherid crabs: (1) apparently in contrast to other decapods, ovaries of pinnotherids can extend from the cephalothorax into the abdomen, thus creating additional space for egg accumulation; (2) the protected habitat allows a reduced calcification of the exoskeleton, which in turn diminishes female body weight and makes the body more flexible, allowing to distend during egg accumulation. The high RO values of pinnotherid crabs are an adaptation to its symbiotic life style (Hines 1992). Our results regarding *Austinotheres angelicus* provide further evidence that pinnotherids can produce proportionately much larger broods than free-living crabs.

### Relation between host size and *Austinotheres angelicus*

The size of *Austinotheres angelicus* increased significantly with the volume of its host, *Saccostrea palmula*. This finding concurs with results reported by Sun et al. (2006) who found that CWs of the pea crab *Pinnotheres sinenis* Shen, 1932 increased with the weight of both the shell and the soft tissue of its host *Mytilus galloprovincialis*. Also, Atkins (1926) found the largest individuals of *Pinnotheres pisum* (Linnaeus, 1767) in large-sized specimens of its host mussel, *Mytilus edulis*.

## Conclusions

Representatives of the family Pinnotheridae have evolved a series of adaptations to cope with their symbiotic life style (Hines 1992; Peiró and Mantelatto 2011), which makes them an interesting model to study the evolution of associations between decapods and other invertebrates. Here we present results on reproductive features of *Austinotheres angelicus*, which lives in association with oysters. Since the host plays a fundamental role in the life cycle of the pea crab, it seems necessary to get a better understanding of the interactions between population dynamics of the host and adaptive responses of the symbiotic pea crab. Hernández et al. (2012) assumed that small host populations induce pea crabs to adopt a solitary life style, and predicted that stable dwellings stimulate extended parental care. Moreover, the larval phase and recruitment processes are unknown in *Austinotheres angelicus*, and it has been speculated that pinnotherid larvae suffer higher mortality rates while searching for the specialized recruitment place (different types of hosts) than free-living species (Lardies and Castilla 2001). Finally, *Austinotheres angelicus* has been reported as symbiont from different hosts (Campos 2002), which raises the question if populations of this pea crab develop different life cycle adaptations in different hosts.
